# Roles of socioeconomic status, ethnicity and teacher beliefs in academic grading

**DOI:** 10.1111/bjep.12541

**Published:** 2022-08-23

**Authors:** Lewis Doyle, Matthew J. Easterbrook, Peter R. Harris

**Affiliations:** ^1^ University of Sussex Brighton UK

**Keywords:** educational inequality, ethnicity, meritocracy, socioeconomic status, teacher bias

## Abstract

**Background:**

Educational outcomes in the United Kingdom vary as a function of students' family background, with those of lower socioeconomic status (SES) and certain ethnic minority groups among the worst affected.

**Aims:**

This pre‐registered study investigates: (i) whether knowledge about students' socioeconomic and ethnic background influences teachers' judgements about the quality of their work and potential for the future, and (ii) the role of teachers' beliefs—most notably about meritocracy—in their practices.

**Sample:**

Our findings are based on the responses of 416 in‐service (88%) and trainee (12%) teachers who successfully passed several stringent exclusion criteria.

**Methods:**

As part of a 2 × 2 independent measures design, teachers were randomly assigned to assess an identical piece of work ostensibly written by a student who varied by SES (higher vs. lower) and ethnicity (White British vs. Black Caribbean). Following this, they responded to several measures assessing their beliefs about education.

**Results:**

Teachers judged students of lower SES to be inferior to students of higher SES across a range of indicators. By contrast, we found no evidence of racial bias in teachers' judgements, though potential reasons for this are discussed. Teachers who believed that schooling is meritocratic were significantly less likely to support equity‐enhancing teaching practices and initiatives.

**Conclusions:**

Unconscious teacher biases and beliefs may be contributing to the relative underperformance of students from poorer backgrounds. These findings provide a mandate for educational institutions to help teachers reflect upon, and develop the skills required to mitigate potentially harmful biases.

## BACKGROUND

Education is associated with a cascade of benefits in terms of employment, fulfilment, wealth, social support and sense of control—all of which contribute to greater overall health and life expectancy (Hahn & Chattopadhyay, 2019; Krueger et al., 2015; Ross & Wu, 1995). However, educational outcomes in the United Kingdom vary as a function of students' family background. In 2020, students who were eligible for free school meals (FSM)—often used as a proxy measure of socioeconomic status (SES; Taylor, 2018)—were only around half as likely to achieve a strong pass (grades 9–5, formerly A*‐C) in GCSE English and maths as their non‐FSM‐eligible peers (Department for Education, 2021a, 2021b). In the same year, the rate of strong passes among Black Caribbean students (34.8%) was fifteen per cent lower than the national average (49.9%). Moreover, whereas 53.7% of non‐FSM White British students met this strong pass standard, only 38.5% of non‐FSM Black Caribbean students did, suggesting that inequality is not restricted to those of low SES (Gillborn et al., 2012). Many of these pre‐existing inequalities are likely to have become exacerbated by the COVID‐19 pandemic (Anders et al., 2021; Easterbrook et al., 2022; Education Endowment Foundation, 2020; Goudeau et al., 2021), thus underlining the timeliness and urgency of research into this area.

In recent years, a wealth of social psychological research has shown the potential of low‐cost, student‐targeted interventions to reduce the existing gap in attainment. These interventions, such as values affirmations (Cohen et al., 2006; Hadden et al., 2020), social belonging manipulations (Shnabel et al., 2013; Walton & Cohen, 2007) and growth mindset training (Dweck & Yeager, 2019) aim to alter the subjective experiences of students by addressing how they make sense of themselves and the world around them (Easterbrook & Hadden, 2020; Walton & Yeager, 2020). However, there is a growing recognition that the success of these ‘wise’ interventions—so‐called because they embody a holistic view of stigmatized individuals (Goffman, 1963)—depends on the characteristics of the local educational context. Indeed, in contexts where social psychological processes do not contribute to educational inequalities, or that lack a fertile supportive environment in which the ‘seeds’ of the intervention can grow, the intervention will likely fail (Binning & Browman, 2020; Easterbrook & Hadden, 2020; Sherman et al., 2021; Walton & Yeager, 2020). Evidently, an environmental change may often be required for lasting improvements in educational equality. This study will investigate how the judgements and practices of teachers—an integral component of the educational environment—may be influenced by unconscious biases and beliefs.

Unconscious processes and biases are automatic, energy‐saving, widespread and often fundamental aspects of human nature (Greenwald & Banaji, 1995; Kahneman, 2011; Lai & Banaji, 2020; Macrae et al., 1994). However, in school settings, despite the best intentions of well‐meaning teachers, unconscious biases may also bear significant costs for students from negatively stereotyped backgrounds—an issue that is particularly salient and timely due to the necessity for teacher grading of academic qualifications during the COVID‐19 pandemic.

### SES bias

Research suggests that biases relating to SES are ingrained from an early age and are easily activated (Kraus et al., 2019; Shutts et al., 2016). Of note, several studies have shown that teacher judgements can be biased by the social status of their students. For example, teachers have shown an implicit bias in favour of children with higher over lower‐educated parents (Pit‐ten Cate & Glock, 2018) and may be more likely to deem a higher academic track more suitable for high‐SES than low‐SES children, even when performance is equal (Batruch et al., 2019). In another intriguing study by Batruch and colleagues, pre‐service teachers assessed tests completed by high or low‐SES students who were presented as either high or low achieving. Results indicated that teacher evaluation of the high achieving low‐SES student's test was harsher than all others. The authors argue that high achieving low‐SES students threaten the social hierarchy and thus prompt teachers to restore order by downgrading their ability (Batruch et al., 2017). Although the above evidence indicates the existence of SES bias in education, there has not yet been any experimental research on this theme in the United Kingdom.

Existing UK‐focused correlational research has, however, highlighted a link between socioeconomic background and grading. Campbell (2015) analysed data of almost 5000 students from the Millennium Cohort Study and found that income‐level bias contributed to inequalities in teachers' ratings of ability and attainment. Similarly, Anders et al. (2021) found that, during the COVID‐19 pandemic, students with a graduate parent were 15% more likely to be given a better teacher assessed grade than their Ofqual (England's exam watchdog) calculated grade, compared with students whose parents had not graduated from university. This suggests that teachers' judgements were swayed by their students' backgrounds in ways that reinforce existing inequalities.

### Ethnicity bias

Negative implicit biases towards ethnic minorities are likely to be a major contributor to educational inequality due to their automaticity and pervasiveness (Warikoo et al., 2016). Research in the United States suggests that—as is the case in the general population—most teachers hold a pro‐White implicit bias (Starck et al., 2020). Indeed, when rating students' academic achievement, behaviour and ability based on a vignette, teachers tend to show a bias in favour of students with typically white‐sounding over Black‐sounding names (Anderson‐Clark et al., 2008). Moreover, a meta‐analysis by Tenenbaum and Ruck (2007) revealed that US‐based teachers had higher expectations for and spoke more positively towards, European American than African American or Latinx students. Although recent research has highlighted the promise of same‐race teacher–student dyads in mitigating these biases (Gershenson et al., 2018; Lindsay & Hart, 2017), pro‐white bias has also been found in the judgements of ethnic minority teachers—an effect which could potentially be attributed to internalized stereotypes (Copur‐Gencturk et al., 2020).

These issues extend beyond American schools, with a number of correlational and qualitative studies revealing that UK‐based Black Caribbean students are systematically under‐rated (Burgess & Greaves, 2013; Campbell, 2015), subject to lower academic expectations (Gillborn et al., 2012), misallocated to lower sets (Connolly et al., 2019) and under‐represented in the higher tiers of maths and science exams, even after controlling for a range of factors such as prior attainment, SES, family circumstances and neighbourhood deprivation (Strand, 2012). However, despite these findings, there has been a dearth of experimental studies into teacher ethnicity bias in the United Kingdom. As such, this study will add to the existing literature by using experimental methods in a UK setting to explore the roles of students' socioeconomic and ethnic backgrounds on teachers' judgements.

Hypothesis 1 Teachers' judgements in assessment will be biased by the SES and ethnicity of their students, with teachers giving higher ratings to students of:

- Higher (vs. Lower) SES.
- White British (vs. Black Caribbean) ethnicity.

### Belief in school meritocracy as a moderator

Conceptualisations of meritocracy posit that individuals reap what their effort and ability deserve. This belief forms the basis of the American dream (Kluegel & Smith, 1986) and has been adopted by many other cultures as a dominant ideology (Kim & Choi, 2017). As such, meritocracy is held up as a bastion of hope for those in the lower echelons of society to upwardly mobilise. However, due to the unequal starting points from which we begin, children from richer families are afforded greater access to enriching home environments and support from parents who have the knowledge, motivation and confidence to help with their learning than their less advantaged peers (Easterbrook et al., 2022). Equally, children from minority ethnic groups are more likely to face psychologically damaging racial discrimination than their majority ethnic peers, which can be detrimental to their academic confidence (Chapman & Bhopal, 2019). In sum, the meritocratic principle may not accurately reflect the varying gradients that children must climb in order to succeed.

Research suggests that the adoption of meritocratic ideals in education may paradoxically serve to maintain inequality by rewarding the privileged, whilst justifying a culture of blame towards less advantaged students, even for realities far beyond their control (Hoyt et al., 2021; Kuppens et al., 2018; Mijs, 2016; Milner, 2010; Reay, 2020; Ullucci, 2007). Moreover, greater belief in meritocratic worldviews is linked to more racist attitudes (Glover, 1994; Katz & Hass, 1988), denial of racial inequity (Knowles & Lowery, 2012), greater stigmatization of minoritized groups (Begue & Bastounis, 2003), more internal attributions for poverty (Godfrey & Wolf, 2016), reduced support for affirmative action (Augoustinos et al., 2005) and lower engagement in methods that promote educational equality (Darnon et al., 2018). In sum, if these pervasive beliefs are held by teachers, this may generate unfair treatment of their students. Consequently, this study will add to previous literature by investigating the links between teachers' beliefs in school meritocracy and their teaching practices.

Hypothesis 2 Teachers with stronger belief in school meritocracy (BSM) will give lower ratings to students from lower SES and Black Caribbean groups than teachers with lower levels of BSM.

Hypothesis 3 Higher levels of BSM will be associated with less support for the following equity‐enhancing initiatives:

- Affirmative action.
- Contextual university offers.
- Teaching practices that accommodate the needs of a diverse range of students.

### Present study

This pre‐registered study^1^ uses both experimental and correlational methods in a UK setting to investigate the influence of student characteristics on teachers' assessment judgements, and the extent to which teachers' beliefs—most notably surrounding the concept of meritocracy—relate to their practices.

## METHODS

Ethical approval was obtained from the institutional ethics board.

### Sample

We used G*Power to conduct a power analysis with the goal of obtaining .80 power to detect an interaction effect size of *f* ^2^ = .0363 at the .05 alpha error probability. The expected effect size was taken from main effects in existing literature (Batruch et al., 2017) and halved to increase sensitivity to detect interaction effects. The power analysis revealed a target sample size of *N* = 403 after exclusions.

Pre‐ and in‐service teachers (*n* = 937) completed a 15‐min online study about education. Of these, 17 withdrew consent after learning about the study's true aims. Additionally, to ensure that our sample was attentive to the key manipulation and subsequent questions, we pre‐registered several stringent exclusion criteria. Data were excluded if participants failed at least one of two manipulation checks about the target's SES (*n* = 154) and ethnicity (*n* = 203),^2^ failed at least one of two attention checks (*n* = 232), spent less than 7 min^3^ on the entire study (*n* = 9) or for a combination of these reasons. The resultant final sample comprised 416 teachers (*M*_age_ = 36.62, *SD* = 10.63; *M*_experience_ = 9.97, *SD =* 9.21; 88% in‐service teachers, 84.1% female, 94.5% White, 3% Asian or mixed White and Asian, <1% all other ethnicities). This sample falls broadly in line with the current teacher workforce in England, which features over‐representation of females and those from a White background (75.8% and 91%, respectively; Department for Education, 2021a, 2021b).^4^

### Design

For the experimental hypotheses (1 & 2), participants were randomly allocated to one of four groups as part of a 2 (SES: Higher vs. Lower) × 2 (Ethnicity: White British vs. Black Caribbean) independent measures design. Hypothesis 3 used correlational analysis to test the strength of the relationship between BSM and support for initiatives that may benefit historically disadvantaged student groups.

### Procedure

Participant teachers in England and Wales were recruited via social media posts, and emails to schools and teacher training centres. After reading an information page, they consented to take part in two ostensibly separate online studies about education on the Qualtrics platform. Participants were randomly allocated to one of four manipulation conditions and informed that the first study would be about how confidentiality and assessment vary internationally. This included the completion of a bogus data protection task and assessment of a short piece of written work. Following this, participants immediately began the second study, which was about views on education and contained the battery of scale measures listed below. Finally, participants provided their demographic details before being fully debriefed on the study's true aims, given the opportunity to withdraw their data, and offered the chance to enter a prize draw for a £150 donation to their school.

### Manipulation

Teachers were randomly allocated to read the student record of one of 4 year six (age 10–11) students whose profiles were manipulated to vary by SES (higher or lower) and ethnicity (White British or Black Caribbean). Similar to the study by Batruch et al. (2017), SES was manipulated by several subtle cues, such as the student's first name (typically high vs. typically low SES) and parental occupation (e.g., doctor vs. cleaner), but we also included a field indicating eligibility for FSM (No vs. Yes), and an indication of extra‐curricular activities (e.g., going skiing with family in the alps vs. playing football with children from the estate). Ethnicity was also manipulated via student's name and an ethnicity section on the form (Black Caribbean or White British). Teachers were informed that the student attended a state primary school with average student demographics and attainment. As a method of encouraging participants to engage with this information, they were presented with a bogus task about confidentiality, in which they were asked to highlight parts of their student's school record that would be considered a confidentiality breach to share with people who were not directly involved with the child's education or welfare. Two manipulation checks for the target's SES and ethnicity were included at the end of the study to confirm that participants had been sensitive to the manipulation: ‘Relative to other people in his school, would you say that the student and his family are in the upper or lower half in terms of income distribution?’ and ‘Which of the following best describes the student's ethnic background?’ (Asian, Arab, Black or White).

#### Main outcomes: grading task

For the main manipulation outcome, teachers in all four conditions were then asked to provide judgements on an identical piece of handwritten work, ostensibly written at the end of year 6 by the student whose profile they had just read. In reality, the writing was that of a genuine year 6 student, which had featured in an end of Key Stage 2 (10–11‐year olds) English writing standardization exercise as an example of the expected standard (see Supplementary Materials; Standards and Testing Agency, 2019). Participants graded the written work from 1 (poor) to 10 (excellent), allocated the target student to an English set based on their ability and potential from 1 (top) to 5 (bottom; these were later reverse‐coded such that higher numbers indicated a more advanced ability grouping), reported how many objective errors there were (0–20) and decided whether the student was working at, below or above the expected standard for their age.

### Moderating, secondary and exploratory variables

We used the following measures to assess how a number of other policy‐relevant attitudes and beliefs related to teachers' practices. In each case, constructs were created as a mean score of the combined items:

#### Belief in school meritocracy

The extent to which teachers believed that schooling is meritocratic was measured by implementing the 8‐item belief in school meritocracy scale (Wiederkehr et al., 2015). Participants were asked to what extent they agreed with items such as ‘At school, students who obtain poor grades are those who have not worked enough’ on a 7‐point scale ranging from 1. Not at all to 7. Very much, with higher scores indicating a stronger belief that schooling is meritocratic. Reliability was acceptable after removing two items^5^ (α = .73), and the remaining items were combined to create a mean composite score.

#### Malleability of intelligence

To assess the extent to which teachers believed that intelligence is fixed (vs. capable of growing), participants answered eight items from Dweck's Mindset Instrument (Dweck, 1999; Dweck et al., 1995) including ‘You can learn new things, but you can't really change your basic intelligence’. Responses were given on a 6‐point scale (1. Strongly disagree to 6. Strongly agree; α = .94).

#### Affirmative action

Participants were asked about their views on affirmative action for ethnic minorities and people living in poverty using six items adapted from Jacobson (1985). Items included ‘I favour affirmative action programmes in education for people of ethnic minorities’ and were measured on a 5‐point scale (1. Strongly Disagree to 5. Strongly Agree), with higher scores indicating greater support for affirmative action. Items for ethnic minorities and people living in poverty were correlated (α = .75), and were thus combined for analysis.

#### Professional beliefs about diversity

To assess teachers' beliefs about representation, diversity and teaching practices, the survey included an adapted version of Pohan and Aguilar's (2001) professional beliefs about diversity scale. The 13‐item scale included items about supporting students from ethnic minority and lower SES backgrounds, such as ‘the traditional classroom has been set up to support the middle‐class lifestyle’ (1. Strongly disagree to 5. Strongly agree; α = .70).

#### Contextual offers

A new 3‐item scale asked teachers the extent to which they agreed with offering candidates from certain groups reduced grade requirements to access higher education courses (i.e., ‘It is not fair to offer different university entry criteria for different people’ [Reversed]). Responses were obtained on a 5‐point scale from 1. Strongly disagree to 5. Strongly agree (α = .87).

#### Political orientation

Participants were asked to what extent they considered themselves to be right or left‐wing on both economic and social issues on a 7‐point scale. Items were highly correlated (α = .86) and, therefore, combined.

## RESULTS

Overall means and those by treatment condition are presented in Table 1 and indicate that across the grade, set and level measures, teachers descriptively rated students of higher SES more favourably than their lower SES peers. By contrast, descriptive ratings of White British and Black Caribbean students were similar across outcomes. Table 1 also shows differences in variation across the outcomes (see appendices 1–4 in the Supplementary Materials for frequency distributions). To test the influence of student SES and ethnicity on teachers' judgements, we specified two models for each dependent variable: overall grade, set allocation, level and number of objective errors. In model 1, each outcome was regressed on student SES (coded 0 for higher SES and 1 for lower SES) and ethnicity (White British = 0, Black Caribbean = 1) using a robust linear model in R.^6^ For model 2, we also included the two‐way interaction between SES and ethnicity. Model parameters and their associated *p*‐values are in Table 2.

**TABLE 1:** Means and standard deviations, overall and by condition

	Group	Overall		White British		Black Caribbean	
		*M*	*SD*	*M*	*SD*	*M*	*SD*
Grade	Overall	6.86	1.33	6.82	1.38	6.90	1.27
	Higher SES	7.04	1.37	7.07	1.33	7.01	1.41
	Lower SES	6.66	1.26	6.55	1.38	6.78	1.10
Set (R)	Overall	3.61	.82	3.65	.81	3.58	.84
	Higher SES	3.75	.87	3.78	.87	3.70	.88
	Lower SES	3.48	.74	3.50	.70	3.45	.78
Level	Overall	1.90	.62	1.91	.63	1.89	.61
	Higher SES	2.04	.63	2.09	.63	1.98	.64
	Lower SES	1.75	.57	1.70	.57	1.80	.58
Errors	Overall	7.47	4.20	7.39	4.11	7.56	4.32
	Higher SES	7.44	4.12	7.16	3.93	7.78	4.34
	Lower SES	7.51	4.30	7.66	4.31	7.33	4.30

**TABLE 2:** Model parameters and associated *p*‐values

Predictor (by outcome)	Model 1					Model 2					Model 3				
	Est	SE	95% CIs	*t*	*p*	Est	SE	95% CIs	*t*	*p*	Est	SE	95% CIs	*t*	*p*
***Grade***															
Low SES	−.44	.15	−.66, −.14	−2.85	**.005**	−.62	.20	−.86, −.13	−3.03	**.002**	−.44	.15	−.63, −.13	−3.00	**.003**
Black Caribbean	.05	.15	−.14, .35	.31	.755	−.16	.21	−.43, .30	−.76	.447	.05	.15	−.17, .34	.32	.753
Low SES*Black Caribbean	–	–	–	–	–	.38	.31	−.21, .82	1.24	.216	–	–	–	–	–
Low SES*BSM	–	–	–	–	–	–	–	–	–	–	.10	.15	−.19, .31	.66	.508
Black Caribbean*BSM	–	–	–	–	–	–	–	–	–	–	–	–	–	–	–
Low SES*Black Caribbean*BSM	–	–	–	–	–	–	–	–	–	–	–	–	–	–	–
***Set (R)***															
Low SES	−.28	.09	−.43, −.12	−3.09	**<.001**	−.28	.12	−.50, −.08	−2.26	**.024**	−.30	.09	−.41, −.11	−3.51	**<.001**
Black Caribbean	−.05	.09	−.23, .09	−.58	.562	−.05	.12	−.34, .16	−.42	.674	−.06	.09	−.24, .09	−.72	.472
Low SES*Black Caribbean	–	–	–	–	–	−.01	.18	−.29, .36	−.02	.986	–	–	–	–	–
Low SES*BSM	–	–	–	–	–	–	–	–	–	–	.07	.09	−.08, .23	.74	.460
Black Caribbean*BSM	–	–	–	–	–	–	–	–	–	–	–	–	–	–	–
Low SES*Black Caribbean*BSM	–	–	–	–	–	–	–	–	–	–	–	–	–	–	–
***Level***															
Low SES	−.29	.06	−.41, −.17	−4.89	**<.001**	−.39	.08	−.55, −.22	−4.83	**<.001**	−.29	.06	−.40, −.18	−4.88	**<.001**
Black Caribbean	−.01	.06	−.12, .10	−.15	.882	−.11	.08	−.28, .07	−1.34	.181	−.01	.06	−.13, .10	−.18	.858
Low SES*Black Caribbean	–	–	–	–	–	.21	.12	−.02, .46	1.78	.077	–	–	–	–	–
Low SES*BSM	–	–	–	–	–	–	–	–	–	–	.08	.06	−.04, .19	1.32	.187
Black Caribbean*BSM	–	–	–	–	–	–	–	–	–	–	–	–	–	–	–
Low SES*Black Caribbean*BSM	–	–	–	–	–	–	–	–	–	–	–	–	–	–	–
***Errors***															
Low SES	−.27	.36	−.71, .96	−.77	.444	.10	.50	−.52, 1.64	.21	.835	−.28	.38	−.71, .86	−.75	.452
Black Caribbean	.18	.36	−.64, .97	.52	.605	.58	.48	−.42, 1.81	1.18	.239	.16	.38	−.62, 1.04	.41	.680
Low SES*Black Caribbean	–	–	–	–	–	−.81	.72	−2.73, .62	−1.13	.260	–	–	–	–	–
Low SES*BSM	–	–	–	–	–	–	–	–	–	–	.04	.39	−.69, 1.05	.11	.915
Black Caribbean*BSM	–	–	–	–	–	–	–	–	–	–	–	–	–	–	–
Low SES*Black Caribbean*BSM	–	–	–	–	–	–	–	–	–	–	–	–	–	–	–

Predictor (by outcome)	Model 4					Model 5				
	Est	SE	95% CIs	*t*	*p*	Est	SE	95% CIs	*t*	*p*
***Grade***										
Low SES	−.43	.14	−.63, −.13	−2.99	**.003**	−.63	.20	−.86, −.14	−3.20	**.001**
Black Caribbean	.05	.15	−.18, .34	.36	.721	−.16	.21	−.43, .34	−.77	.439
Low SES*Black Caribbean	–	–	–	–	–	.39	.29	−.23, .79	1.33	.184
Low SES*BSM	–	–	–	–	–	.21	.21	−.22, .50	.99	.323
Black Caribbean*BSM	−.15	.15	−.37, .13	−.96	.336	−.03	.21	−.39, .34	−.12	.903
Low SES*Black Caribbean*BSM	–	–	–	–	–	−.22	.30	−.67, .35	−.74	.461
***Set (R)***										
Low SES	−.30	.08	−.43, −.11	−3.53	**<.001**	−.31	.11	−.49, .09	−2.68	**.008**
Black Caribbean	−.06	.08	−.22, .09	−.72	.473	−.06	.12	−.32, .16	−.54	.593
Low SES*Black Caribbean	–	–	–	–	–	.01	.17	−.28, .35	.03	.973
Low SES*BSM	–	–	–	–	–	.27	.12	.05, .47	2.21	**.028**
Black Caribbean*BSM	−.06	.09	−.22, .11	−.63	.530	.17	.12	−.10, .38	1.34	.180
Low SES*Black Caribbean*BSM	–	–	–	–	–	−.43	.18	−.70, −.05	−2.40	**.017**
***Level***										
Low SES	−.29	.06	−.40, −.17	−4.85	**<.001**	−.38	.08	−.53, −.24	−4.79	**<.001**
Black Caribbean	−.01	.06	−.12, .11	−.15	.884	−.11	.08	−.27, .07	−1.36	.176
Low SES*Black Caribbean	–	–	–	–	–	.21	.12	−.05, .44	1.75	.081
Low SES*BSM	–	–	–	–	–	.15	.08	−.01, .30	1.80	.072
Black Caribbean*BSM	−.03	.06	−.17, .08	−.54	.589	.05	.09	−.14, .24	.55	.581
Low SES*Black Caribbean*BSM	–	–	–	–	–	−.16	.12	−.42, .09	−1.25	.210
***Errors***										
Low SES	−.27	.37	−.78, .84	−.74	.462	.10	.48	−.58, 1.46	.21	.835
Black Caribbean	.18	.37	−.66, 1.01	.48	.632	.53	.49	−.54, 1.77	1.07	.285
Low SES*Black Caribbean	–	–	–	–	–	−.77	.72	−2.60, .73	−1.08	.282
Low SES*BSM	–	–	–	–	–	−.35	.52	−1.53, .85	−.67	.502
Black Caribbean*BSM	.12	.39	−.15, 1.45	.30	.765	−.22	.52	−1.11, 1.45	−.42	.675
Low SES*Black Caribbean*BSM	–	–	–	–	–	.78	.74	−.63, 2.87	1.05	.294

*Notes*: 1. Model 1: SES + Ethnicity; Model 2: SES × Ethnicity; Model 3: SES + Ethnicity + (SES × BSM); Model 4: SES + Ethnicity + (Ethnicity × BSM; Model 5: SES × Ethnicity × BSM).

2. SES was coded 0 = Higher SES (ref.) and 1 = Lower SES. Ethnicity was coded 0 = White British (ref.) and 1 = Black Caribbean.

3. The BSM variable was mean‐centred in all models.

4. The Set variable was reverse‐coded such that higher scores indicate a more favourable judgement.

5. Results are reported with 95% bias‐corrected and accelerated confidence intervals (BCa CIs) with 2000 random bootstrap samples.

6. *p* values in bold are significant at at least the *p* < .05 level.

### Overall grade

**FIGURE 1:**
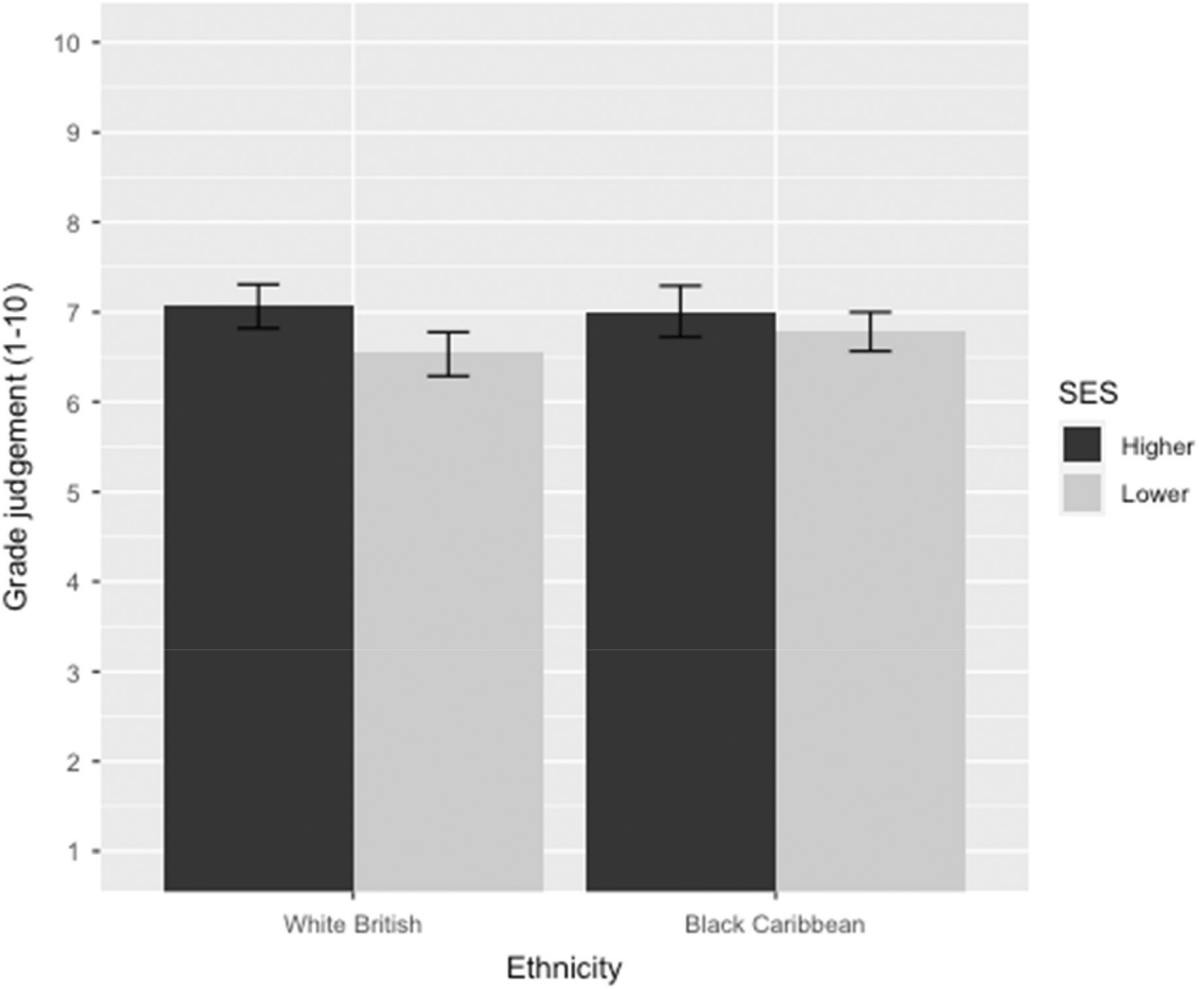
Teacher grade judgement as a function of student SES and ethnicity. Error bars represent 95% confidence intervals.

In support of hypothesis 1a, teachers shown the work of a student from a lower SES background were significantly harsher in their grading compared to those who were shown the work of a student of higher SES (*p* < .01; η^2^ = .02; see Figure 1). This equates to a grading difference of 4.4% between those of higher and lower SES. However, there was no significant main effect of ethnicity on overall grade (*p* = .755). The two‐way interaction between SES and ethnicity was also non‐significant (*p* = .216).

### Set allocation

**FIGURE 2:**
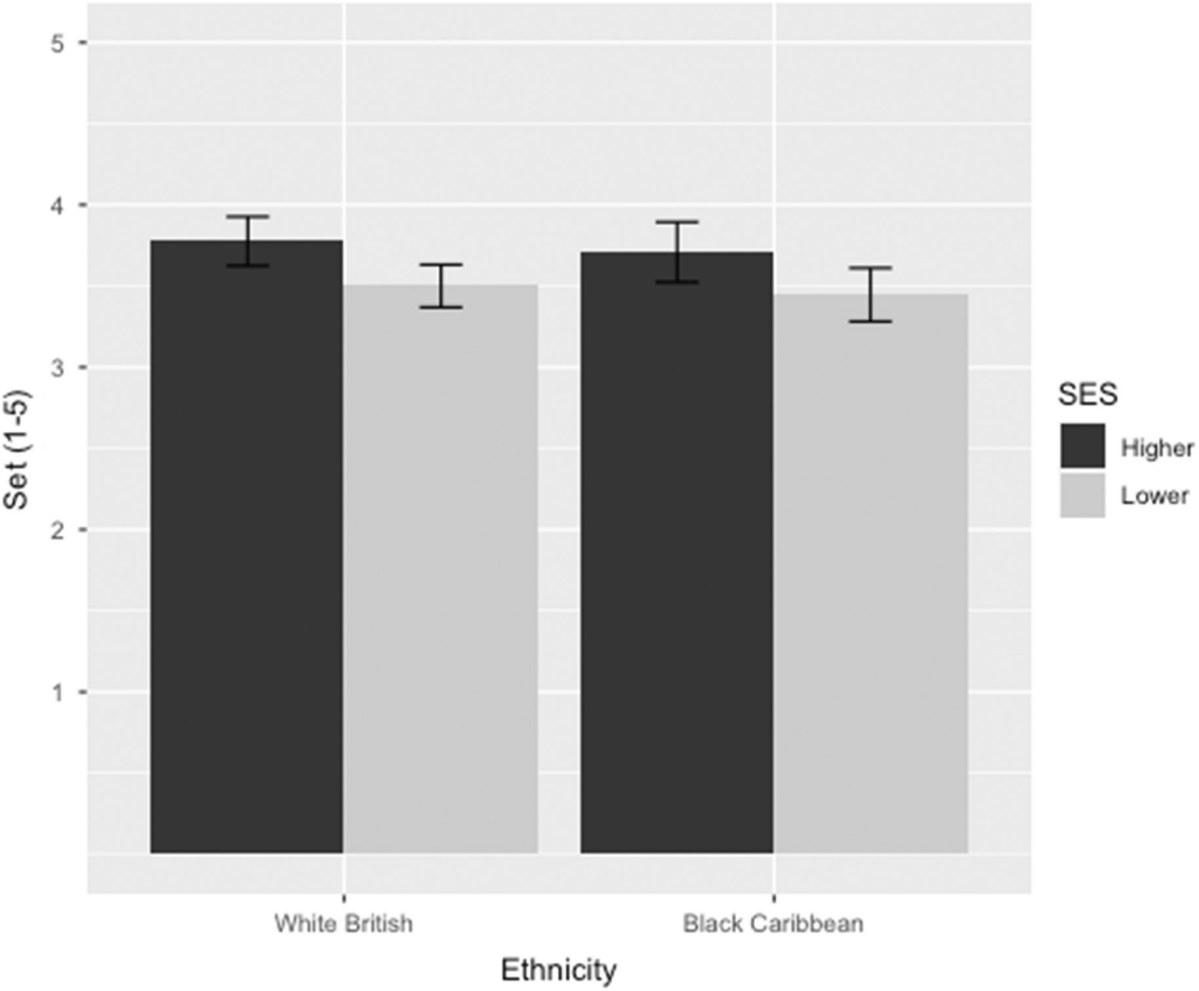
Teacher set allocations as a function of student SES and ethnicity. Scores were reverse‐coded such that higher numbers indicate a higher ability grouping. Error bars represent 95% confidence intervals.

For ease of interpretability, set allocation scores were reversed so that higher numbers indicate a more advanced ability grouping. The SES of the target student significantly predicted teachers' set allocations. Supporting hypothesis 1a, students of lower SES were allocated to lower sets than were those from higher SES backgrounds (*p* < .001; η^2^ = .03; Figure 2). However, student ethnicity did not predict set allocation (*p* = .562), nor was there a significant interaction between SES and ethnicity (*p* = .986).

### Perceived level

**FIGURE 3:**
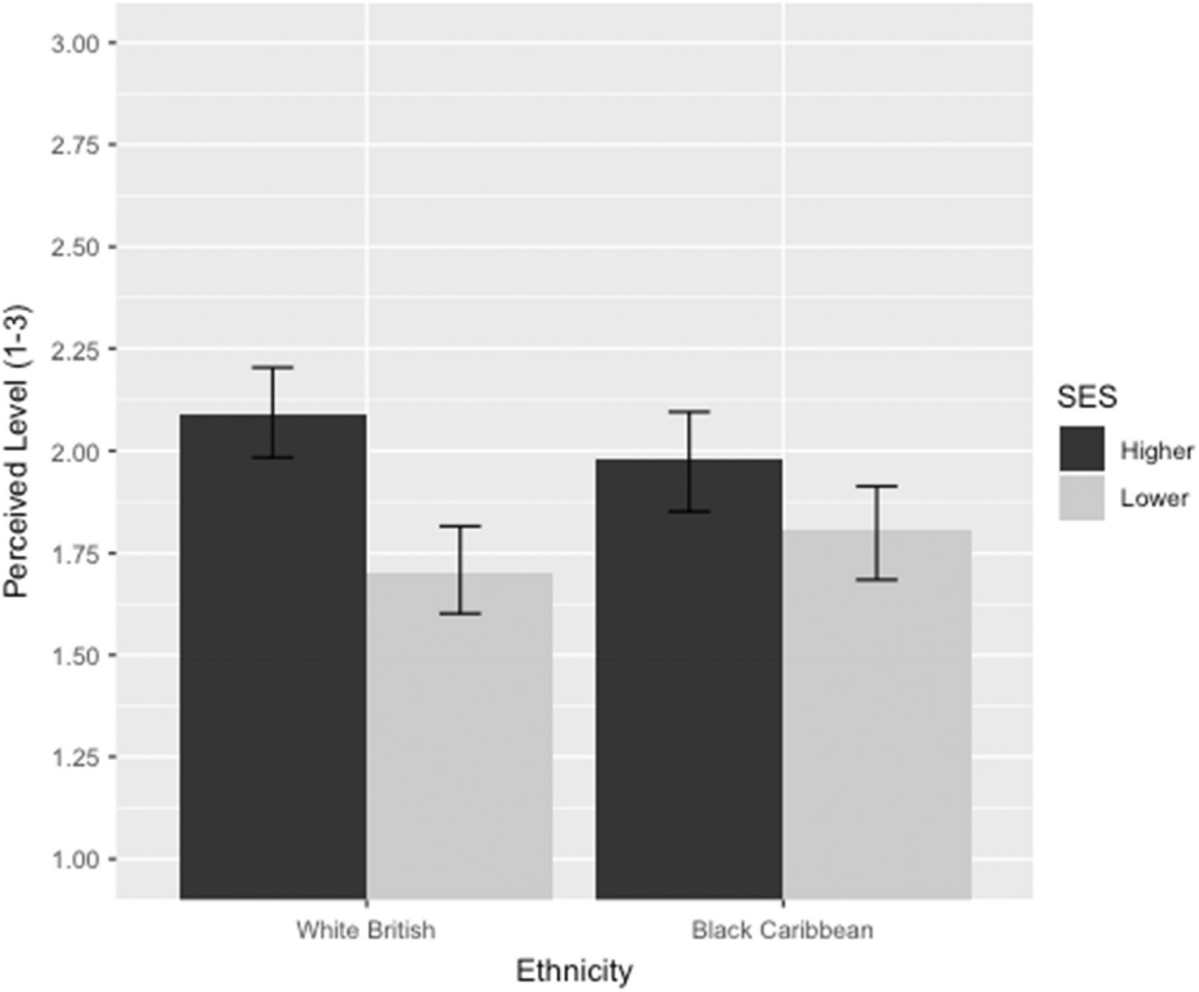
Teacher perceptions of student level as a function of student SES and ethnicity. 1 = Working below the expected level, 2 = Working at the expected level, 3 = Working above the expected level. Error bars represent 95% confidence intervals.

There was a significant main effect of SES on teachers' judgements of current academic level. In support of hypothesis 1a, teachers perceived students of lower SES to be working at an inferior level to those from higher SES backgrounds (*p* < .001; η^2^ = .06; Figure 3). However, as the level outcome only had three values, we also ran a multinomial logistic regression using the multinom function in the nnet package in R (Ripley et al., 2016). In line with the linear model results, the odds of being perceived to be working at (vs. below: *OR* = .58, *p* = .021) or above (vs. below: *OR* = .18, *p* < .001) the expected standard were significantly greater for a student of higher (vs. lower) SES (see Table 3). There was no main effect of ethnicity (*p*s > .8 for both methods of analysis), but there was a marginal two‐way interaction between SES and Ethnicity (*p*s < .1). Using the afex package in R (Singmann et al., 2015), we obtained simple effects of estimated marginal means, revealing that the effect of SES on perceived level was stronger when the target's ethnicity was white British [*F*(1, 412) = 23.35, *p* < .001] than Black Caribbean [*F*(1, 412) = 3.86, *p* = .050].

**TABLE 3:** Model parameters and associated *p*‐values for multinomial logit. (BSM mean‐centred) Reference category = working below expected level

	Predictor	Model 1			Model 2			Model 3			Model 4			Model 5		
		OR	95% CIs	*p*	OR	95% CIs	*p*	OR	95% CIs	*p*	OR	95% CIs	*p*	OR	95% CIs	*p*
*Working at the expected level*	Intercept	3.25	2.15, 4.90	< .001	3.84	2.31, 6.36	<.001	3.28	2.17, 4.96	<.001	3.28	2.16, 4.95	<.001	3.89	2.34, 6.49	<.001
	Low SES	.58	.36, .92	**.021**	.44	.23, .84	.**012**	.59	.36, .94	**.027**	.58	.36, .93	**.023**	.46	.24, .88	**.019**
	Black Caribbean	1.04	.66, 1.66	.857	.73	.36, 1.49	.388	1.03	.65, 1.65	.898	1.03	.65	1.64	.72	.35, 1.48	.369
	Low SES*Black Caribbean	–	–	–	1.82	.71, 4.67	.215	–	–	–	–	–	–	1.75	.68, 4.55	.248
	Low SES*BSM	–	–	–	–	–	–	1.63	.99, 2.68	.056	–	–	–	1.87	.92, 3.79	.082
	Black Caribbean*BSM	–	–	–	–	–	–	–	–	–	.80	.49, 1.29	.356	1.02	.48, 2.12	.955
	Low SES*Black Caribbean*BSM	–	–	–	–	–	–	–	–	–	–	–	–	.73	.27, 1.98	.532
*Working above expected level*	Intercept	1.27	.77, 2.11	.352	1.58	.89, 2.80	.119	1.28	.77, 2.13	.342	1.28	.77, 2.13	.335	1.60	.90, 2.86	.112
	Low SES	.18	.09, .36	**< .001**	.10	.04, .28	**<.001**	.18	.09, .37	**<.001**	.18	.09, .37	**<.001**	.10	.03, .29	**<.001**
	Black Caribbean	.93	.48, 1.79	.820	.57	.24, 1.34	.198	.92	.48, 1.78	.803	.91	.47, 1.76	.783	.55	.23, 1.32	.183
	Low SES*Black Caribbean	–	–	–	3.42	.80, 14.59	.097	–	–	–	–	–	–	3.42	.78, 15.03	.103
	Low SES*BSM	–	–	–	–	–	–	1.39	.65, 2.96	.393	–	–	–	2.25	.73, 6.89	.157
	Black Caribbean*BSM	–	–	–	–	–	–	–	–	–	.88	.44, 1.76	.723	1.32	.53, 3.26	.551
	Low SES*Black Caribbean*BSM	–	–	–	–	–	–	–	–	–	–	–	–	.378	.08, 1.77	.216

*Notes*: 1. Model 1: SES + Ethnicity; Model 2: SES × Ethnicity; Model 3: SES + Ethnicity + (SES × BSM); Model 4: SES + Ethnicity + (Ethnicity × BSM; Model 5: SES × Ethnicity × BSM). 2. SES was coded 0 = Higher SES (ref.) and 1 = Lower SES. Ethnicity was coded 0 = White British (ref.) and 1 = Black Caribbean. 3. The BSM variable was mean‐centred in all models. 4. *p* values in bold are significant at at least the *p* < .05 level.

### Number of objective errors

**FIGURE 4:**
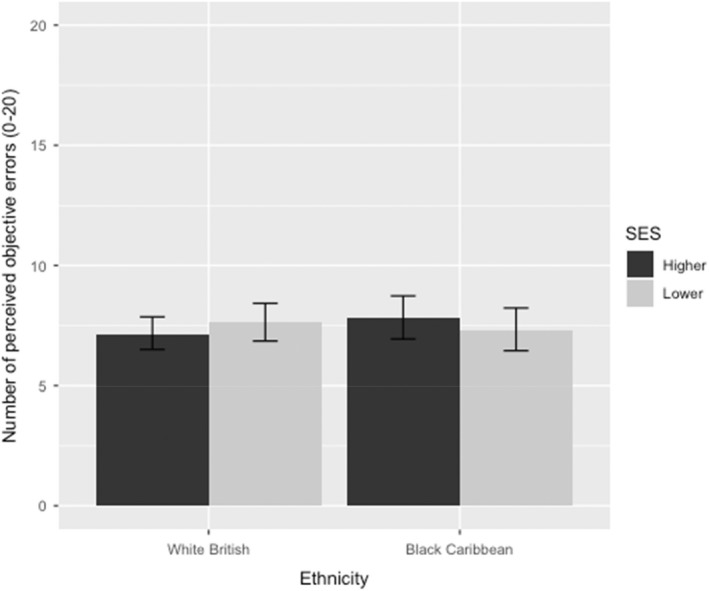
Teacher perceptions of the number of objective errors as a function of student SES and ethnicity. Error bars represent 95% confidence intervals.

There were no significant main effects of SES or ethnicity on teacher judgements of objective errors (*p*s > .4; Figure 4), nor was there a two‐way interaction (*p* = .260).^7^

### BSM as a moderator

We tested hypothesis 2 via three models for each outcome. Firstly, model 3 regressed the outcome variable on SES, Ethnicity and the two‐way interaction between SES and BSM, which had been mean‐centred. Model 4 included the main predictors and the two‐way interaction between Ethnicity and BSM. Finally, model 5 included all predictors, two‐way interactions, and a three‐way interaction between SES, ethnicity and BSM (see Table 2).

**FIGURE 5:**
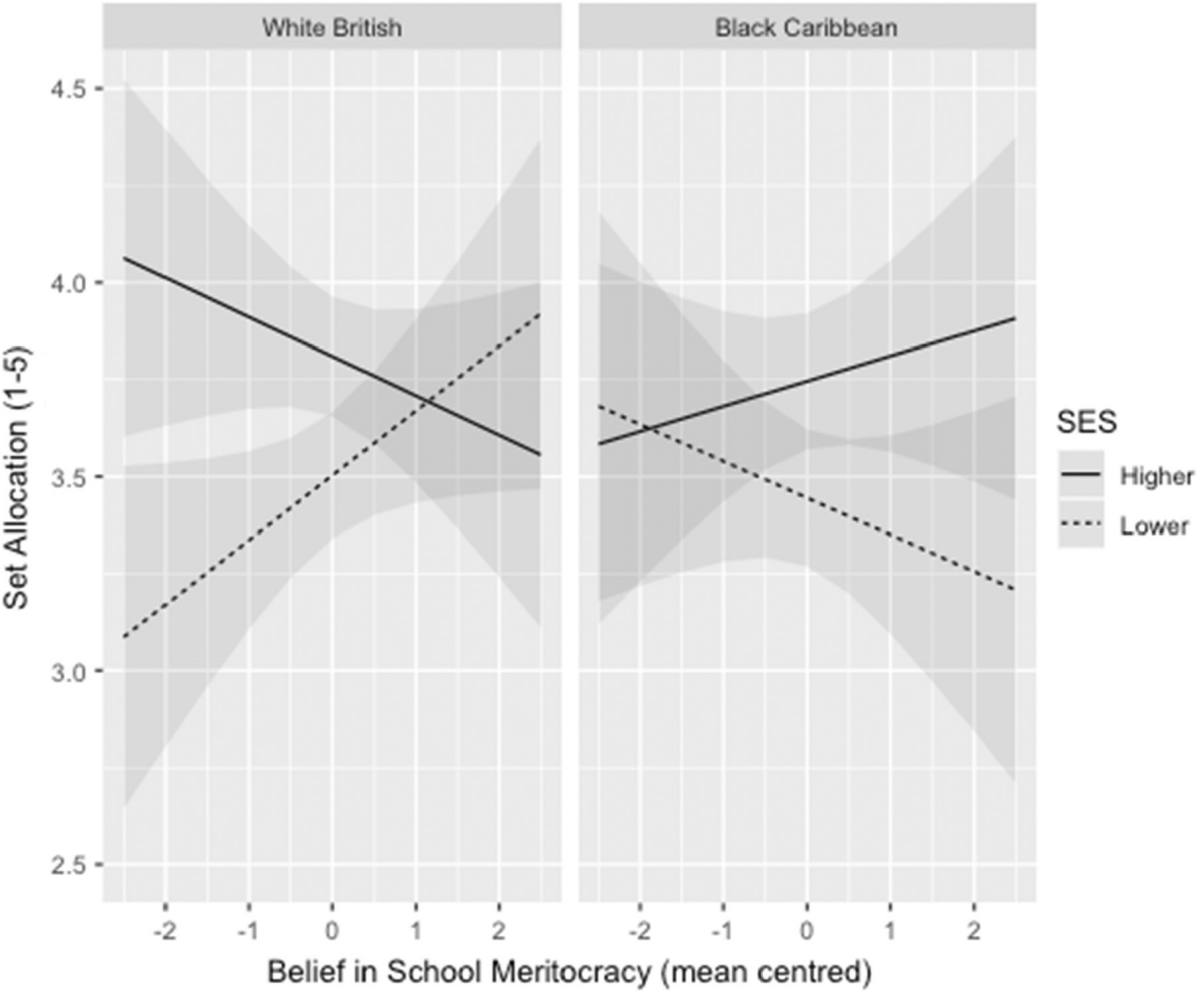
Three‐way interactions between student ethnicity and SES, teachers' belief in school meritocracy for set allocation. The set variable was reverse‐coded such that higher scores indicate a higher ability grouping. Shaded areas represent 95% confidence intervals.

There were no two or three‐way interactions with BSM for the overall grade, level or errors variables (all *p*s > .2). However, model 5 for the set allocation variable revealed a significant two‐way interaction between SES and BSM (*p* = .028), and a significant three‐way interaction between student SES, ethnicity and BSM (*p* = .017, η^2^ = .01). To unpack this interaction, we ran simple effects analyses on each of the four groups (Lower SES, Higher SES, White British and Black Caribbean). As demonstrated in Figure 5, for White British students, there was a significant interaction between SES and BSM, *B* = .25 (.04, .47), *SE* = .11, *t* = 2.30, *p* = .022. This effect was most evident at low levels of BSM, where students from higher socioeconomic backgrounds were allocated to higher sets than those of lower SES. This difference was cancelled out at high levels of BSM, thus going against Hypothesis 2. For Black Caribbean students, there was no significant interaction between SES and BSM (*p >* .2). Among lower SES students, there was a significant interaction between ethnicity and BSM, *B =* −.25 (−.46, −.04), *SE* = .11, *t* = −2.31, *p* = .022, whereby stronger BSM predicted more favourable set allocation for lower SES White British students but less favourable allocation for Black Caribbean students from the same SES background. For higher SES students, there was no significant interaction between Ethnicity and BSM, (*p* > .2).

### BSM and support for equity‐enhancing initiatives

A linear model showed that BSM scores were not significantly predicted by any of the conditions, *F*(3, 412) = .13, *p* = .945, thereby giving us confidence that participants' BSM scores had not been influenced by the earlier tasks. We tested hypothesis 3 by specifying three separate linear models, in which attitudes towards affirmative action, contextualized admissions and diverse teaching practices were separately regressed on belief in school meritocracy. In line with hypothesis 3, teachers' BSM was significantly and negatively associated with support for affirmative action, *B* = −.14 (BCa: −.20, −.07), *SE* = .03, *t* = −4.11, *p* < .001, and teaching practices that accommodate the diverse needs of their students, *B* = −.19 (BCa: −.22, −.13), *SE* = .03, *t* = −7.59, *p* < .001. Moreover, the negative relationship between BSM and contextualized university admissions approached significance, *B* = −.11 (BCa: −.23, .01), *SE* = .06, *t* = 1.84, *p* = .066. These correlational findings suggest that the more teachers believed that schooling is meritocratic, the less they supported initiatives to enhance the development of ethnic minority and low SES students.

### Exploratory analyses

To further probe the relationships between teachers' beliefs and their teaching practices, we added teachers' beliefs about the malleability of intelligence and political beliefs to the robust linear model as predictors along with BSM. Diverse teaching practices were significantly and negatively predicted by BSM, *B* = −.15, *SE* = .03, *t* = −5.84, *p* < .001, teachers' mindsets about the malleability of intelligence, *B* = −.12, *SE* = .03, *t* = −4.39, *p* < .001 and political beliefs, *B* = −.12, *SE* = .02, *t* = −5.81, *p* < .001, with the overall model accounting for 24% of the variance (*R* ^2^ = .24). Thus, stronger beliefs in school meritocracy, fixed mindsets and right‐wing politics predicted less support for equitable teaching practices.

## DISCUSSION

In a well‐powered, pre‐registered study, with a largely representative sample, we found that teachers judged an identical piece of work as being of poorer quality if it was presented as being written by a student of lower rather than higher SES. Based on the written work, teachers also rated lower SES students as having significantly inferior ability and potential (i.e., set allocation), and to be working at a significantly lower overall level compared with higher SES students. Interestingly, there were no significant differences in the number of objective errors identified in the four conditions, thus suggesting that judgements may be biased in spite of objective clues that indicate equal performance.

The absence of any main effects of ethnicity (hypothesis 1b) should be taken with caution, as other evidence suggests that racial biases are prevalent in teacher judgements in the United Kingdom and abroad (Anderson‐Clark et al., 2008; Burgess & Greaves, 2013; Campbell, 2015; Connolly et al., 2019; Copur‐Gencturk et al., 2020; Gillborn et al., 2012; Okonofua & Eberhardt, 2015; Strand, 2012). Our findings cannot be explained by a racial (mis)match in student–teacher dyads, as there were not enough Black teachers in our sample to drive such effects. However, research has suggested that teachers' fears of being perceived as racist may lead them to inflate the grades of Black students (Croft & Schmader, 2012; Nishen & Kessels, 2021), thus mitigating deleterious biases. More simply, it could be that teachers are becoming more aware of ethnicity biases and are thus more likely to monitor and inhibit such influences (e.g., Pope et al., 2018). If true, this would provide hope for the tempering of SES and other biases.

Consistent with Hypothesis 3 and existing literature on the links between meritocracy beliefs and bigoted attitudes, we found that the more strongly teachers believed that schooling is currently meritocratic, the less supportive they were of practices and initiatives aimed at reducing SES and ethnicity‐based inequities. For the most part, our findings did not confirm the prediction that BSM would moderate teachers' judgements. However, we did find partial support in the set allocation outcome for students of lower SES. As predicted, among this group, increasing BSM was associated with inferior set allocations for the Black Caribbean student. However, contrary to predictions, stronger BSM also predicted lower set allocation for the higher SES White British student and higher allocation for the White British student of lower SES. It is notable that only set allocations—with their focus on ability and future potential—yielded a significant interaction and not the outcomes for [current] grade or level. It is possible that the ethnicity‐related differences in set allocations may be linked to the unique negative stereotypes faced by Lower SES Black Caribbean students. Indeed, Priest et al. (2018) found that Black teens were more likely to be perceived as lazy than other ethnic groups. With laziness being the antithesis of the hard work that embodies the meritocratic principle, it is possible that teachers' high in BSM consigned Black Caribbean students of low SES to comparatively lower sets with the expectation that such students would never put in sufficient effort to succeed. More work is needed to add clarity to this theory and the contradictory outcomes for White British students.

### Implications and future research

These findings have clear implications for the education community. Where biases exist, the local educational context is likely to perpetuate rather than attenuate the attainment disparities between students from different backgrounds. Students from marginalized groups may fall prey to a self‐fulfilling prophecy (Rosenthal & Jacobson, 1968) and fear that their contributions in school will confirm negative stereotypes that others hold of them (Steele et al., 2002). However, this should not be deemed an attack on teachers, who often have the unenviable role of facilitating the learning of large groups whilst under a number of other cognitively and emotionally draining pressures. We hereby make several recommendations for educational institutions and researchers.

Firstly, unconscious biases are likely to result from internalized stereotypes and distortions in the world around us, which Tatum (2017) likens to breathing in a ‘smog’ that engulfs society but goes unnoticed without self‐reflection. As such, teachers and trainees alike should be given the time and support to explore their attitudes, beliefs and biases towards marginalized groups. Research in education suggests that these may be somewhat resistant to change (Thompson et al., 2016), but Devine et al. (2012) found that breaking the ‘habit’ of prejudice is possible with motivation and hard work, especially if the individual is aware of their biases, concerned about their consequences, and knows how best to replace them.

Secondly, biases are more likely to be reduced if both the individual and institution are invested in the process (Stephens et al., 2020). As an example, teachers may be more likely to revert to stereotypical or biased judgements at times of high cognitive load (Feldon, 2007) and when there is no clear evaluation rubric (Quinn, 2020). Schools and education boards should work with their teachers to *debias* the learning environment (Murphy et al., 2018) and facilitate practices and workloads that are conducive to equitable teaching.

Thirdly, when confronted with notions of personal biases, teachers' responses may be defensive in nature (Clark & Zygmunt, 2014; Knowles et al., 2014; Solomona et al., 2005). One potential avenue for future research would, therefore, be to engage teachers in a values affirmation (e.g., Cohen & Sherman, 2014; Easterbrook et al., 2021; Steele, 1988). Research has shown that participants who had committed moral transgressions were more likely to repair their wrongdoings if they had been affirmed than those who had not (Wenzel et al., 2020). In theory, affirmations could uncouple the threat teachers may feel when confronted with evidence of their personal transgressions (e.g., exhibiting bias in their grading) from their sense of self (Sherman, 2013), thus making them less defensive and more motivated to change.

Finally, although there was partial support for the BSM moderation hypothesis, the results were not conclusive. Future work should focus on BSM as a moderator of the level of *blame* attributed to poor performance as a function of students' backgrounds. This would add to an existing body of research that suggests strong beliefs in meritocracy predict greater victim blaming (Hoyt et al., 2021; Kuppens et al., 2018).

### Limitations

We were unable to pay participants for their time. As such, many rushed through the study without taking note of the key manipulation and subsequent measures. To counteract these issues, we pre‐registered the inclusion of several stringent manipulation and attention checks to filter out potentially problematic and inattentive participants. As a result, we can be confident that the final sample was attentive and focused to the key manipulations and measures. Nevertheless, removing more than half of the completed (albeit rushed) responses raises an ethical issue of wasted time.

Our results showed a clear and meaningful effect of SES on teachers' judgements, but we did not control for the contexts in which these teachers taught. A replication of this study could measure and control for the amount of contact and experience teachers have with students of lower SES and ethnic minorities.

## CONCLUSION

The findings of this study provide the first UK‐based experimental evidence of teacher bias relating to students' SES, whilst also supporting an existing body of literature on this theme. They also suggest that teachers' beliefs about the meritocratic nature of schooling and the malleability of intelligence are linked to significantly less support for practices and programmes, which aim to support traditionally marginalized student groups.

## AUTHOR CONTRIBUTIONS

**Lewis Doyle:** Conceptualization; data curation; formal analysis; investigation; methodology; project administration; resources; visualization; writing – original draft. **Matthew J. Easterbrook:** Conceptualization; supervision; writing – review and editing. **Peter R. Harris:** Supervision; writing – review and editing.

## CONFLICT OF INTEREST

All authors declare no conflict of interest.

## Supporting information


Appendix S1‐S12

## Data Availability

Data will be made available prior to publication.

## Footnotes

^1^
Pre‐registration: https://osf.io/tpzc9/?view_only=4076750b1a6c4335ab86aad112800eac.

^2^
The probability of passing both manipulation checks by chance was *p* = .125.

^3^
Trial runs indicated the study should take ~14 min to complete. As such, we pre‐registered that we would exclude those who completed the study twice as fast as expected (<7 min).

^4^
The observable characteristics of the final sample were almost identical to the complete sample prior to exclusions, which included 89% in‐service teachers, 84% female, 92% White, 3% Asian or mixed White and Asian, <1% all other ethnicities.

^5^
The items removed were: ‘At school, when there is a will, there is a way’ and ‘Willingness is not always enough to succeed at school’.

^6^
For the level outcome, we report findings from a general linear model with 95% bootstrapped confidence intervals as the robust model would not run.

^7^
We also pre‐registered a secondary hypothesis that teachers who exhibit fixed mindset beliefs would give lower grades to students of low SES and Black Caribbean ethnicity than teachers with growth mindset beliefs. However, there were no significant two or three‐way interactions between teachers' mindsets and SES or ethnicity on any of the judgement outcomes (all *p*s > .2).
