# Supplementary material for: Roles of socioeconomic status, ethnicity and teacher beliefs in academic grading
Source: Br J Educ Psychol. 2022 Aug 23;93(1):91–112. doi: 10.1111/bjep.12541 (PMC10087759; doi:10.1111/bjep.12541)

**Supplementary materials for**

**“The roles of socioeconmic status, ethnicity, and teacher beliefs in academic grading”**

**Appendix 1:**

Frequency distribution for Grade outcome variable


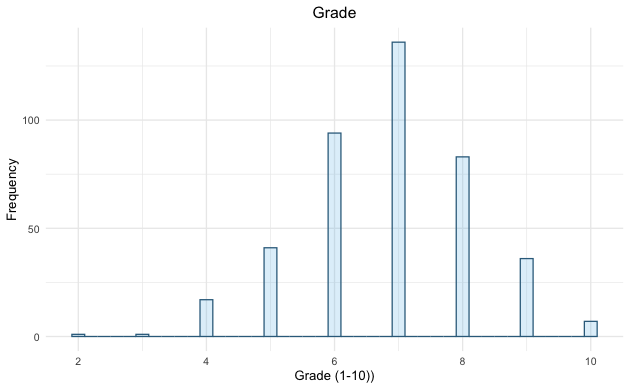


**Appendix 2:**

Frequency distribution for Set Allocation outcome variable (Reversed)

*Note: Higher scores indicate a higher set allocation (greater ability and potential)*


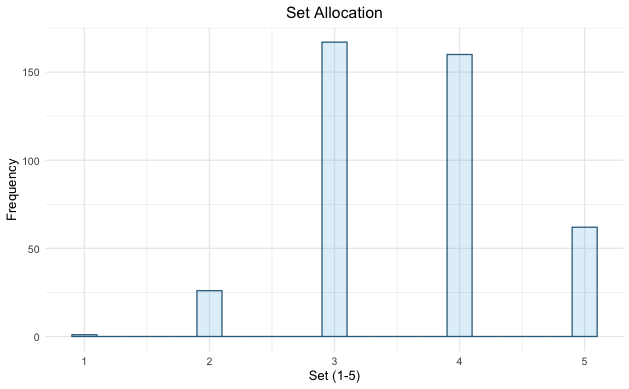


**Appendix 3:**

Frequency distribution for Level outcome variable

*Note: 1 = Working below the expected standard, 2 = Working at the expected standard, 3 = Working above the expected standard.*


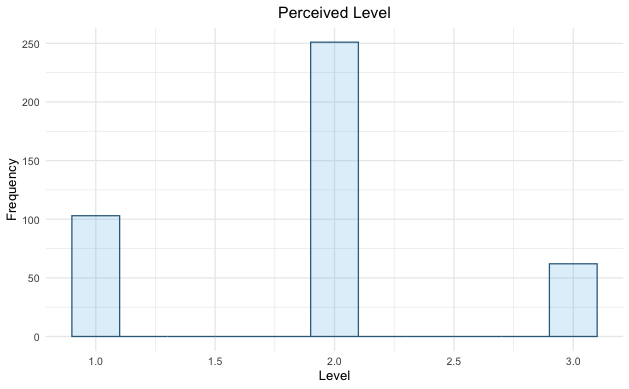


**Appendix 4:**

Frequency distribution for Errors outcome variable


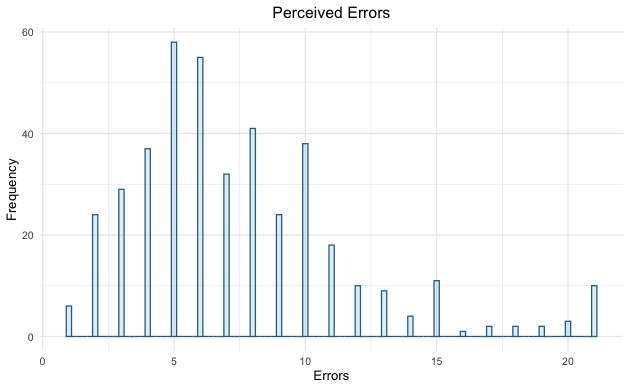


**Appendix 5:**

Frequency distribution for Belief in School Meritocracy scale

*Note: Higher scores indicate stronger belief in school meritocracy.*


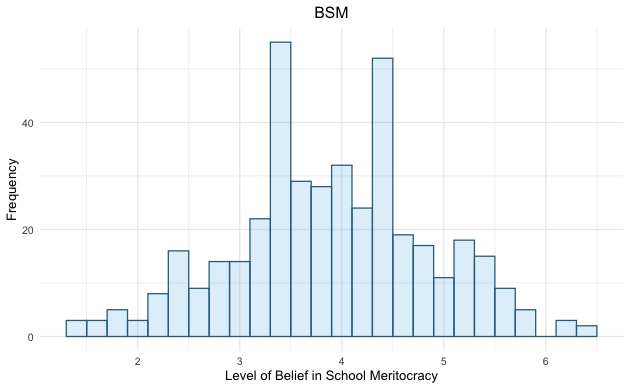


**Appendix 6:**

Frequency distribution for diverse teaching practices

*Note: Higher scores indicate greater support for diverse teaching practices.*


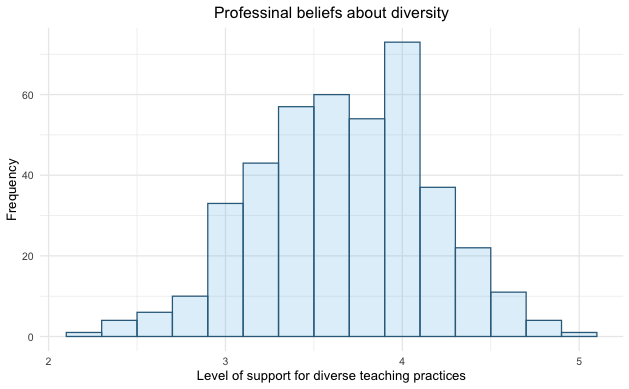


**Appendix 7:**

Frequency distribution for Contextualised University Admissions.

*Note: Higher scores indicate greater support for contextual admissions.*


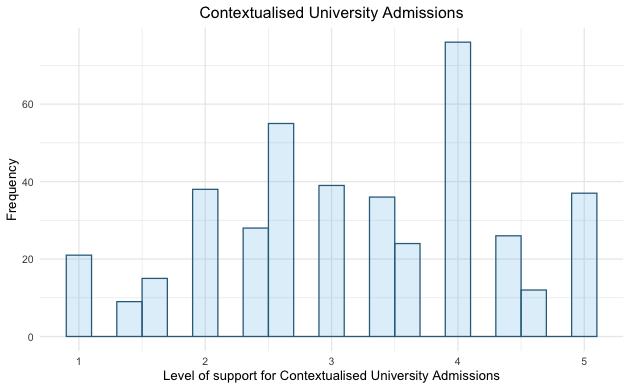


**Appendix 8:**

Frequency distribution for Affirmative Action.

*Note*: *Higher scores indicate greater support for affirmative action.*


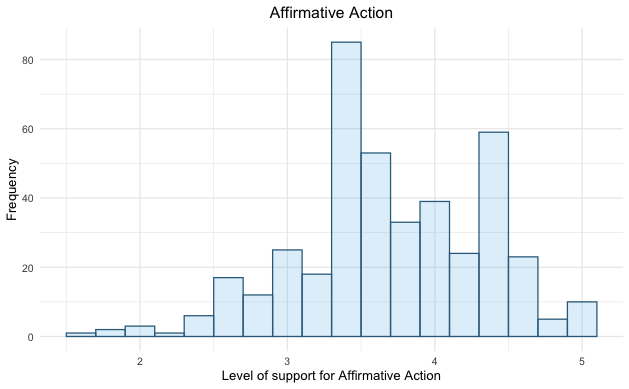


**Appendix 9:**

Frequency distribution for Dweck mindset scale

*Note: Lower scores indicate a growth mindset about intelligence, higher scores indicate a more fixed mindset.*


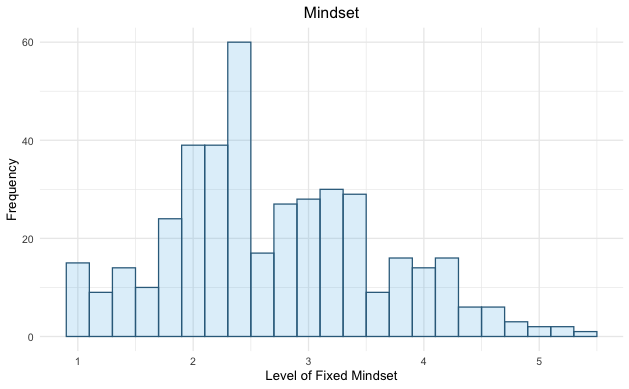


**Appendix 10:**

Scale items and their individual responses

*Note: Mean scores for reversed items are displayed in their original (non-reversed) form.*

Scales

| Scale | Introduction | Item | *M* | *SD* |
| --- | --- | --- | --- | --- |
| Affirmative Action  *(1. Strongly disagree - 5. Strongly agree)* | ***Affirmative action****(also known as positive action) refers to a set of policies and practices within a government or organisation which aim to increase representation in employment or education of people from certain groups, based on characteristics such as race, ethnicity, gender, sexual orientation, physical ability and socioeconomic background.* | *1. After years of discrimination, it is only fair to set up special programmes to make sure that people of ethnic minorities are given every chance to have equal opportunities in education* | 4.10 | .89 |
|  |  | *2. People of ethnic minorities should make it strictly on merit.* (Reversed) | 3.08 | 1.14 |
|  |  | *3. I favour affirmative action programmes in education for people of ethnic minorities* | 3.75 | .88 |
|  |  | *4. After years of discrimination, it is only fair to set up special programmes to make sure that people who live in poverty are given every chance to have equal opportunities in education* | 4.40 | .75 |
|  |  | *5. People living in poverty should make it strictly on merit.* (Reversed) | 2.91 | 1.13 |
|  |  | *6. I favour affirmative action programmes in education for people living in poverty* | 4.03 | .84 |
| Contextualised University Admissions  *(1. Strongly disagree - 5. Strongly agree)* | ***Contextual university offers****require lower grades than usual for those from backgrounds which are historically less likely to attend university. For example, a course that usually requires the grades BBB for entry may be accessible with the grades BCC for students from certain backgrounds.* | *1. Lowering the admission criteria for people from backgrounds that are historically underrepresented in higher education is fair.* | 3.10 | 1.13 |
|  |  | *2. People from backgrounds that are historically underrepresented in higher education should be encouraged to study at university by using contextual offers.* | 3.49 | 1.13 |
|  |  | *3. It is not fair to offer different university entry criteria for different people.* (Reversed) | 3.00 | 1.30 |
| Political Orientation |  | *1. In general, how left-wing (Liberal) or right-wing (Conservative) are you on economic issues?* | 3.03 | 1.36 |
| *(1. Very left-wing – 7. Very right-wing) Responses for “Don’t know/Not political”, and “Can’t pick a label” removed* |  | *2. In general, how left-wing (Liberal) or right-wing (Conservative) are you on social issues (eg. social inequality, criminal justice system, welfare)?* | 2.63 | 1.32 |
| Professional beliefs about diversity  *(1. Strongly disagree - 5. Strongly agree)* |  | *1. Teachers should not be expected to adjust their preferred mode of instruction to accommodate the needs of all students* (Reversed) | 1.75 | 1.02 |
|  |  | *2. The traditional classroom has been set up to support the middle-class lifestyle* | 3.54 | 1.07 |
|  |  | *3. Only schools serving students from historically marginalised ethnic groups need a racially, ethnically, and culturally diverse staff and faculty* (Reversed) | 1.34 | .76 |
|  |  | *4. People of ethnic minorities are adequately represented in most textbooks and learning resources today.* (Reversed) | 2.16 | 1.16 |
|  |  | *5. Generally, teachers should group students by ability levels* (Reversed) | 2.63 | 1.09 |
|  |  | *6. Historically, education has been monocultural, reflecting only one reality and has been biased toward the dominant (White British) group.* | 4.10 | .86 |
|  |  | *7. Teachers often expect less from students from the lower socioeconomic class* | 3.06 | 1.21 |
|  |  | *8. Multicultural education is most beneficial for students from ethnic minority backgrounds.* (Reversed) | 2.16 | 1.17 |
|  |  | *9. In order to be effective with all students, teachers should have experience working with students from diverse racial and ethnic backgrounds.* | 3.51 | 1.10 |
|  |  | *10. Disproportionately large numbers of students from ethnic minority backgrounds are improperly placed in lower sets by school personnel.* | 2.94 | 1.02 |
|  |  | *11. Disproportionately large numbers of students from low socioeconomic backgrounds are improperly placed in lower sets by school personnel.* | 3.05 | 1.05 |
|  |  | *12. Students from lower socioeconomic backgrounds typically have fewer educational opportunities than their middle-class peers.* | 4.03 | .98 |
|  |  | *13. Multicultural education is less important than reading, writing, arithmetic, and computer literacy.* (Reversed) | 2.46 | 1.01 |
| Belief in School Meritocracy  *(1. Not at all - 7. Very much)*  *Note: Items 1 and 8 were removed to improve scale reliability.* |  | *1. At school, when there is a will, there is a way* | 5.06 | 1.42 |
|  |  | *2. Everyone has the same chances to succeed at school* | 3.58 | 1.78 |
|  |  | *3. To succeed at school, one only has to work hard* | 3.78 | 1.67 |
|  |  | *4. At school, students who obtain poor grades are those who have not worked enough* | 2.50 | 1.32 |
|  |  | *5. At school, students are rewarded (they obtain good grades, praise) for their efforts* | 5.25 | 1.23 |
|  |  | *6. At school, children obtain the grades they deserve* | 4.11 | 1.34 |
|  |  | *7. At school, students who obtain good grades are those who have worked hard* | 4.22 | 1.41 |
|  |  | *8. Willingness is not always enough to succeed at school* (Reversed) | 5.11 | 1.36 |
| Dweck Mindset Scale  *(1. Strongly disagree - 6. Strongly agree)* |  | *1. You have a certain amount of intelligence, and you can’t really do much to change it.* | 2.45 | 1.03 |
|  |  | *2. Your intelligence is something about you that you can’t change very much* | 2.41 | 1.04 |
|  |  | *3. No matter who you are, you can significantly change your intelligence level* (Reversed) | 4.26 | 1.03 |
|  |  | *4. To be honest, you can’t really change how intelligent you are* | 2.40 | 1.03 |
|  |  | *5. You can always substantially change how intelligent you are* (Reversed) | 3.96 | 1.12 |
|  |  | *6. You can learn new things, but you can’t really change your basic intelligence* | 2.88 | 1.20 |
|  |  | *7. No matter how much intelligence you have, you can always change it quite a bit* (Reversed) | 4.18 | 1.03 |
|  |  | *8. You can change even your basic intelligence level considerably* (Reversed) | 4.10 | 1.09 |
|  |  |  |  |  |

**Appendix 11:**

Manipulation: Student Records

i) Higher SES / White British


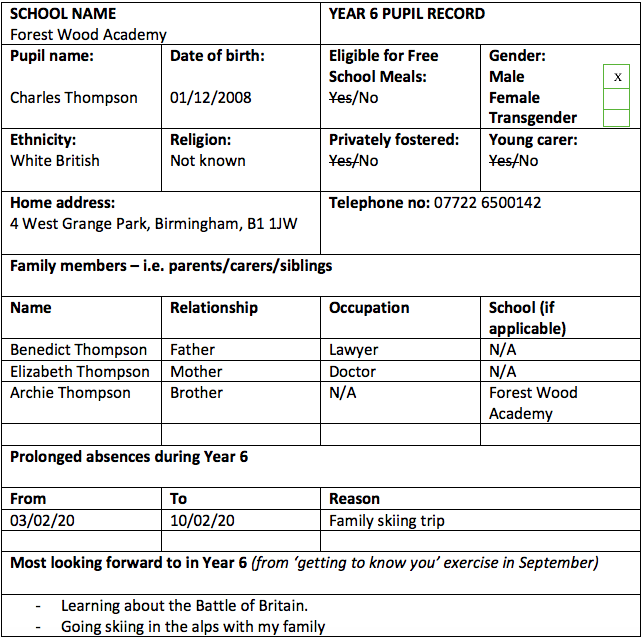


ii) Lower SES / White British


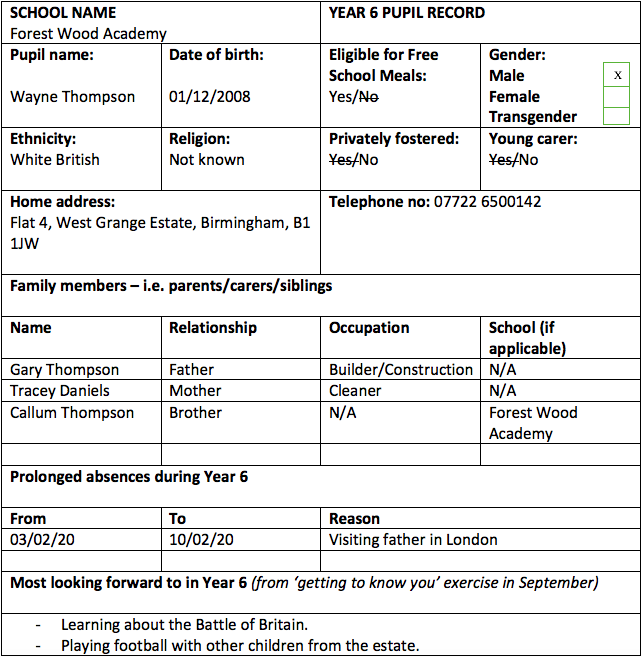


iii) Higher SES / Black Caribbean


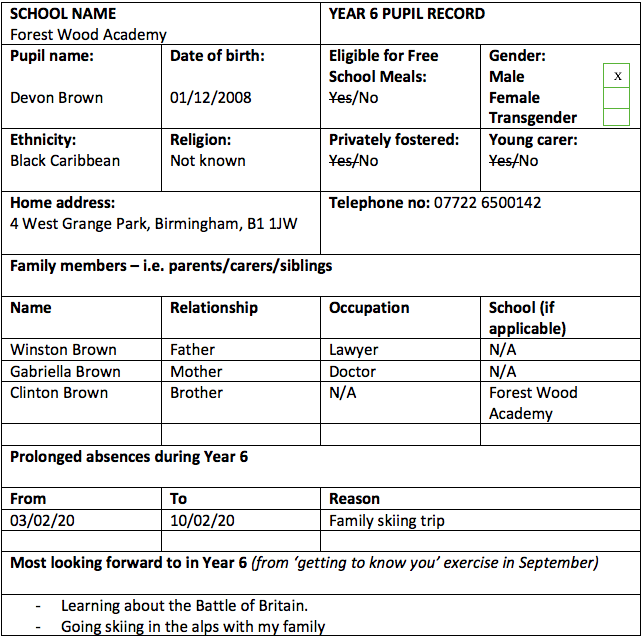


iv) Lower SES / Black Caribbean


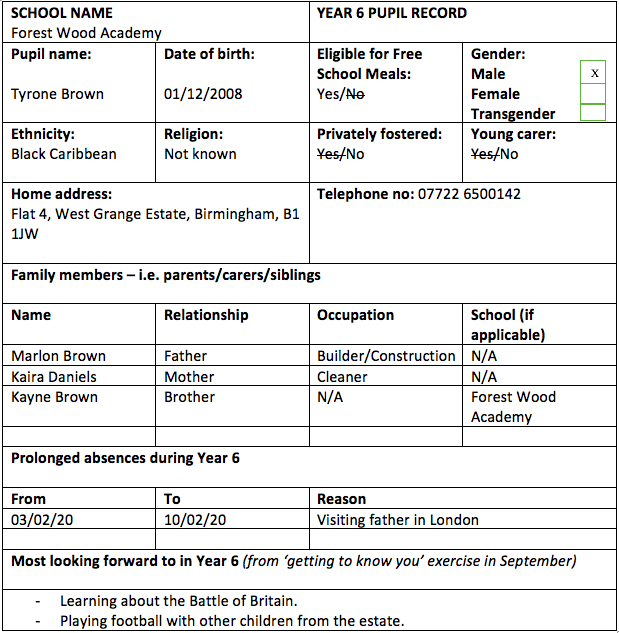


**Appendix 12:**

Year 6 handwritten work


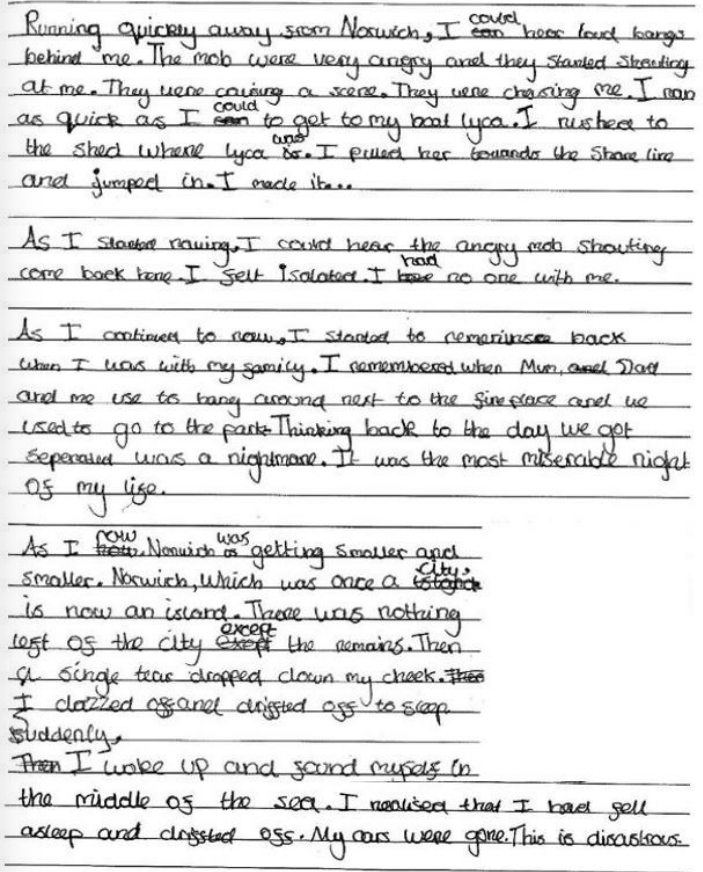

Supplement: Supplementary file 1 — Appendix S1‐S12 [file BJEP-93-91-s001.docx]
